# The Southern California Extracorporeal Membrane Oxygenation Consortium During the Coronavirus Disease 2019 Pandemic

**DOI:** 10.1017/dmp.2021.179

**Published:** 2021-06-08

**Authors:** Mazen Odish, Cassia Yi, Juliann Eigner, Amelia Kenner Brininger, Kristi L. Koenig, David Willms, Suzan Lerum, Scott McCaul, Ayana Boyd King, George Sutherland, Lynette Cederquist, Robert L. Owens, Travis Pollema

**Affiliations:** 1 University of California, San Diego Health, San Diego, California, USA; 2 Scripps Health, San Diego, California, USA; 3 County of San Diego Health and Human Services Agency, San Diego, California, USA; 4 Sharp HealthCare, San Diego, California, USA; 5 Rady Children’s Hospital, San Diego, California, USA

**Keywords:** coronavirus, pandemics, extracorporeal membrane oxygenation, United States, resource allocation

## Abstract

In March 2020, at the onset of the coronavirus disease 2019 (COVID-19) pandemic in the United States, the Southern California Extracorporeal Membrane Oxygenation (ECMO) Consortium was formed. The consortium included physicians and coordinators from the 4 ECMO centers in San Diego County. Guidelines were created to ensure that ECMO was delivered equitably and in a resource effective manner across the county during the pandemic. A biomedical ethicist reviewed the guidelines to ensure ECMO use would provide maximal community benefit of this limited resource. The San Diego County Health and Human Services Agency further incorporated the guidelines into its plans for the allocation of scarce resources. The consortium held weekly video conferences to review countywide ECMO capacity (including census and staffing), share data, and discuss clinical practices and difficult cases. Equipment exchanges between ECMO centers maximized regional capacity. From March 1 to November 30, 2020, consortium participants placed 97 patients on ECMO. No eligible patients were denied ECMO due to lack of resources or capacity. The Southern California ECMO Consortium may serve as a model for other communities seeking to optimize ECMO resources during the current COVID-19 or future pandemics.

The coronavirus disease 2019 (COVID-19) pandemic has stressed medical communities across the world and challenged all aspects of surge capacity, including hospital beds, medications, staff, ventilators, and specialized resources such as extracorporeal membrane oxygenation (ECMO).^1,2^ ECMO is an advanced type of life-support that may be used for patients with acute respiratory distress syndrome (ARDS).^3–5^ Although ECMO resources and use have increased in adults over the past decade, notably after the 2009 H1N1 influenza pandemic, it remains a scarce resource not available at all hospitals.^6^ The large number of simultaneous cases of severe ARDS in the COVID-19 pandemic has substantially stretched ECMO resources. Currently, the World Health Organization guidelines recommend transfer to an ECMO center for COVID-19 patients with severe ARDS that is refractory to conventional therapies, but this may not always be possible due to patient instability or resource limitations.^7^ Thus, regional disaster planning should consider ECMO as a scarce, yet vital, resource and plan accordingly.

In the setting of a pandemic, allocation of scarce resources, such as ECMO, must shift from prioritization of the good of the individual to the good of the community when crisis standard of care is declared.^1,8^ The ethical guiding principles in this setting include the prioritization of the most lives saved, equity, transparency, and duty to plan. Establishing crisis standards in advance, will contribute to maximizing the number of lives saved. Triage and allocation must be applied equitably to all patients using objective criteria as much as possible. The decisions for ECMO candidacy must be made by individuals who are not directly caring for patients to minimize moral distress of clinicians and allow them to maintain trust and fidelity to the patients under their care. When crisis standards are declared, patients and families should be fully informed of the criteria upon which decisions are made in advance.^9^

Geographic access to centers with ECMO capabilities varies widely across the United States.^10^ In 2014, approximately 58.5% of the population lived within 1-h driving distance from an ECMO center.^10^ However, this has likely increased as there were only 171 ECMO centers in 2014. Currently, there are approximately 286 ECMO centers, many of which are concentrated in high population areas, with the top 30 largest metropolitan areas containing at least 1 ECMO center.^11,12^

Due to limited access and resources, regional collaboration and operational planning with the goal of equitable ECMO distribution to maximize benefit is warranted. During a COVID-19 pandemic surge, regional partnerships may also help alleviate ad hoc decisions for ECMO allocation. Similar collaborations have been successful in Minnesota where health-care leaders developed a state-wide ECMO inclusion criteria based on ECMO capacity, projected duration of ECMO, and predicted survival.^13^ Herein, we describe the development and maintenance of our San Diego ECMO Consortium, and additionally, report resources used and outcomes.

## Regional Geography and COVID-19 Overview

**Figure 1.**
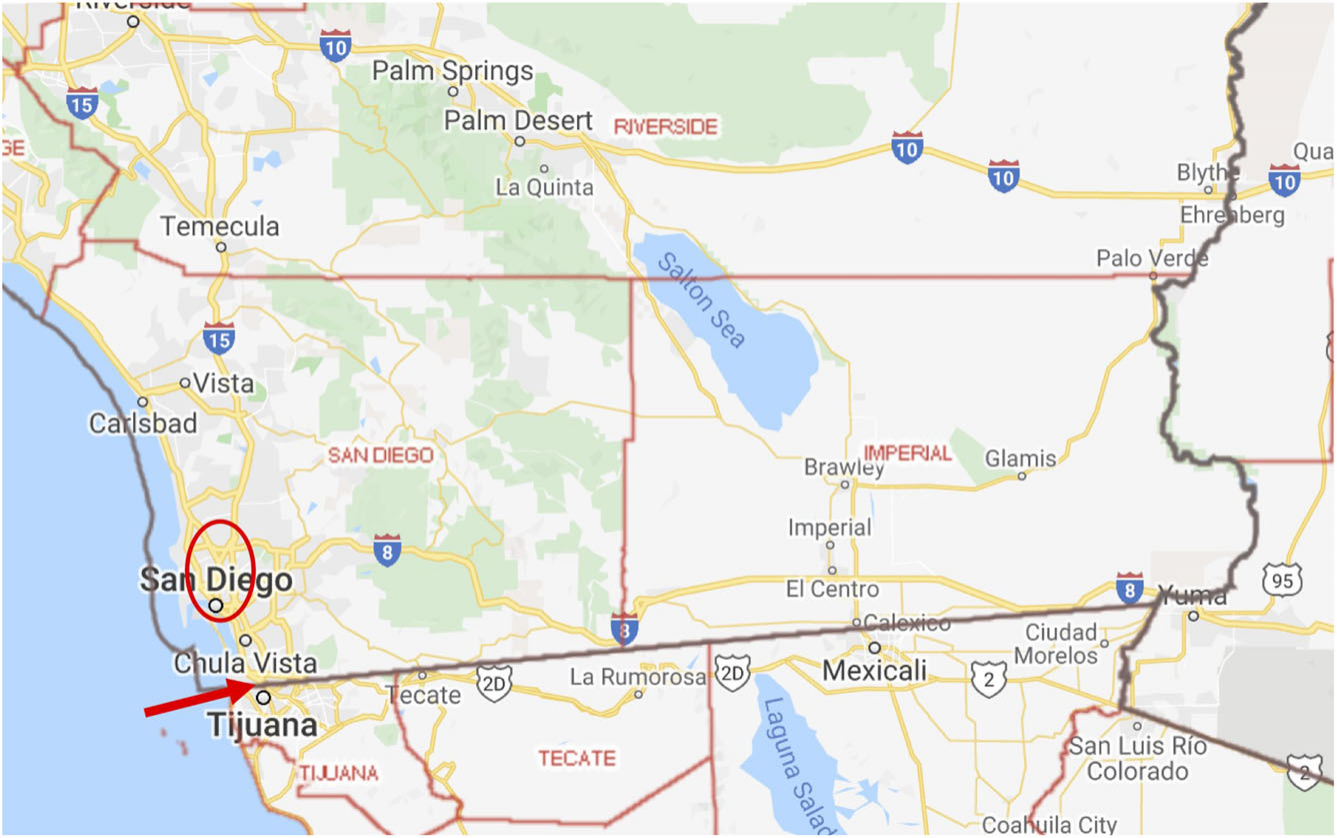
Southern California and Baja California, Mexico. San Diego and Imperial counties located in Southern California, bordering the state of Baja California, Mexico. The city of San Diego borders Tijuana, Mexico while the city of El Centro, California borders Mexicali, Mexico. Arrow, San Ysidro Land Port of Entry. Circle, the location of the 4 ECMO centers (3 adult, 1 pediatric) in San Diego County. Map created by Google Maps, Alphabet Inc, Mountain View, California.

San Diego and Imperial are the southernmost counties in California, located on the border with Mexico (Figure 1).^14^ San Diego County is 4525 square miles with 3.3 million residents, making it the second and fifth most populated county in California and United States, respectfully (Table 1).^15,16^ As of November 30, 2020, there have been 83,421 cases of severe acute respiratory syndrome coronavirus 2 (SARS-CoV-2) in San Diego County; the fourth highest number of cases in California, 27th highest cases per 100,000 (2488.9 per 100,000).^17^ On December 1, 2020, it reported a 7-day average infection rate of 47 per 100,000 (Table 1).^17^ There are 24 acute care hospitals in the San Diego County, 6 of which have the ability to place patients on ECMO support.^18^ However, only 3 centers in the county are able to provide management and comprehensive care for adult patients on ECMO.

**Table 1.** Consortium contengency phases and respective ECMO exclusion criteria

**Conventional phase (not in pandemic)**	
Each ECMO center follows internal criteria for selection.	
**Contigency phase ECMO criteria (<80% county-wide ECMO use)**	
**Relative contraindications**	**Absolute contraindications**
- BMI (>35 kg/m^2^) - Known terminal disease/cancer, life expectancy <5 years - Poor prehospital clinical frailty score - Devastating/major debilitating neurologic injury - Known underlying lung disease that would compromise recovery or on home oxygen - Shock requiring high vasopressor requirement, plus significant hypoxia - Ventilator dependence >10 days - Expected poor prognosis following CPR	- Do not resuscitate - Contraindication to anticoagulation - Cirrhosis with MELD score >30 - Known pre-existing heart failure (EF <35%) - ESRD on outpatient hemodialysis (AKI/ARF is not an exclusion criteria) - >2 acute organ failure, excluding AKI/ARF
**Crisis phase ECMO criteria (≥ 80% county-wide ECMO use)**	
**Relative contraindications**	**Absolute contraindications**
- BMI >30	- Do not resuscitate - Known terminal disease/cancer, life expectancy <5 years - Contraindication to anticoagulation - Cirrhosis with MELD score >30 - Known pre-existing heart failure (EF <35%) - ESRD on outpatient hemodialysis (AKI/ARF is not an exclusion criteria) - Ventilator dependence >10 days - >1 Acute organ failure, excluding AKI/ARF - Known poor prehospital frailty - Devastating/major debilitating neurologic injury - Known underlying lung disease that would compromise recovery or on home oxygen - Expected poor prognosis following CPR

AKI, acute kidney injury; ARF, acute renal failure; BMI, body mass index (kg/m^2^, kilogram per meter squared); CPR, cardiopulmonary resuscitation; DNR, do not resuscitate; EF, ejection fraction; ESRD, end-stage renal disease; MELD, Model for End-Stage Liver Disease.

Imperial County has a population of 179,957, and there have been 16,364 confirmed SARS-CoV-2 cases as of November 30, 2020.^19^ Imperial County is 4,481 square miles, making it larger than the state of Delaware and Rhode Island. Although Imperial County ranks 30th out of the 58 California counties for population, it ranked first in SARS-CoV-2 cumulative incidence (9080.2 cases per 100,000). As of December 1, 2020, Imperial County has reported a daily incidence rate of 52.8 per 100,000 (Table 1).^15,19^ There are only 2 hospitals in Imperial County, which have limited intensive care unit (ICU) beds and no ECMO capabilities; for this reason the consortium agreed to consider Imperial County residents for ECMO.^18^ As a result of limited resources and high infection rates, a substantial amount of patients with COVID-19 have been transferred out of Imperial County to hospitals throughout California since the beginning of the pandemic.^20,21^

When planning for the COVID-19 pandemic in Southern California, an important consideration was proximity to the border with Mexico. Tijuana, Mexico, borders the city of San Diego and has a population of 2.1 million and, as of November 30, 2020, has reported 7963 (379.2 per 100,000) SARS-CoV-2 cases. However, this is likely under-reported due to limited testing capabilities and reports that patients are only tested when admitted to the hospital and not as outpatients.^22,23^ The San Ysidro Land Port of Entry between San Diego and Tijuana is the busiest land border crossing point in the world (Figure 1) with more than 50 million crossings into the United States in 2019.^24,25^ Similarly, Mexicali, the capital city of the State of Baja California, Mexico, borders El Centro, California, the largest city in Imperial County with thousands of border crossings daily.^26^ As of November 30, 2020, Mexicali had the highest total SARS-CoV-2 infections in Mexico with 12,342 (1234.2 per 100,000) cases reported.^27^ Before the pandemic, there were no hospitals in Tijuana or Mexicali that offered ECMO. As of November 30, 2020, 1 patient has been placed on ECMO in a private hospital in Tijuana. This hospital acquired ECMO equipment during the pandemic.

## Methods

### Development and Implementation of the ECMO Consortium

#### Key Stakeholders, ECMO Centers

In March 2020, two ECMO coordinators from different ECMO centers in San Diego championed the consortium, which included medical directors, physicians, and coordinators from all San Diego County ECMO centers. A biomedical ethics expert was included to ensure appropriate ethical principles were followed. Every ECMO center in the county participated, and weekly video conferencing (Zoom application, Zoom Video Communications) meetings were established. After the ECMO Consortium guidelines were developed, they were distributed to the regional hospitals and the San Diego County Health & Human Services Agency (the local public health department).

#### Guideline Goals and Development

The objective of the consortium was to ensure equitable use of ECMO resources throughout the region and unified ECMO criteria for patients with COVID-19 at all the centers. Due to ECMO being resource intensive, it was agreed that if critical care capacity was overwhelmed, then ECMO would not be used until resources stabilized or improved.

The ECMO exclusion criteria were initially based on the Extracorporeal Life Support Organization (ELSO) Guidelines for Adult Respiratory Failure, since at that time their COVID-19 specific guidelines had not yet been published.^28^ The consortium guidelines were adjusted after the ELSO COVID-19 guidelines were released in April and May of 2020.^28,29^ Similar to prior guidelines, the ECMO exclusion criteria was divided into relative and absolute contraindications.

Three ECMO pandemic phases were developed: conventional, contingency, and crisis phase. The phase would be determined by the consortium’s percentage of ECMO resources used (ie, current ECMO census/total ECMO capacity). The consortium’s ECMO criteria would then vary depending on the ECMO pandemic phase (Table 1). Thus, as the percentage of resource use increases, the ECMO selection criteria became more conservative to maximize regional benefit and patient survival. Accordingly, many of the relative ECMO contraindications became absolute contraindications in crisis phase. ECMO capacity was also affected by on ICU bed availability, equipment, and staffing. This assessment of ECMO census and capacity required continuous communication between the consortium centers.

#### Health Department Role in Consortium

San Diego County’s COVID-19 response was guided by the principles and metrics in the All-Hazard Health Services Capacity Management Plan. The plan focuses on patient care capacity and was structured around the 3S System for Surge Capacity: “Staff, Stuff, and Structure.”^1^ ECMO capacity and use data from each center were aggregated to provide a county-wide assessment of ECMO availability and shared with the Health Services Capacity Task Force which is comprised of county administration, hospital and prehospital representatives, and other members of the health care community. This group, in conjunction with the County’s COVID-19 Incident Management Team, reviewed the data daily and met on a regular basis to discuss management of regional health-care capacity, including an assessment of current status and an evaluation of potential emerging shortages. If an increasing trend of San Diego ECMO resource use were to be detected, then steps would be taken to assist the centers to build capacity. This would include requesting additional staffing resources or equipment from surrounding counties or the California Department of Public Health.

### Consortium Guidelines

#### The Conventional Phase

In the conventional ECMO phase (nonpandemic), traditional care was followed. In this phase, each institution used their own internal criteria to assess appropriateness of ECMO use. If patients were deemed borderline candidates, a request for review by the other centers was available as a second opinion.

#### The Contingency Phase

The consortium moved into the contingency phase once the COVID-19 pandemic was declared by the World Health Organization on March 11, 2020.^30^ This phase also requires that less than 80% of the county-wide ECMO resources (including non-COVID-19 patients on ECMO) are being used.^30^ At each individual center, ECMO candidacy was determined by a minimum of 2 physicians with ECMO experience, not directly involved in the patient’s care, using the consortium guidelines.

Two of the consortium centers (Scripps Health and Sharp HealthCare) have the ability to provide ECMO supported cardiorespiratory resuscitation (eCPR) in their emergency departments. During this phase, eCPR was not offered to patients with COVID-19 or patients under investigation. This was due to a paucity of evidence for any survival benefit with eCPR in patients with COVID-19 and to minimize health-care exposures during cannulation.^31^

#### The Crisis Phase

The consortium entered the crisis phase if the county reached 80% of ECMO capacity (including non-COVID-19 patients). In this phase, due to unknown survival benefit, in addition to eCPR, veno-arterial ECMO was no longer offered to any patients with COVID-19. Furthermore, interfacility transfer requests for ECMO were not accepted from hospitals outside of the San Diego County during crisis phase. However, out of county transfers continued between centers with pre-existing agreements and from Imperial County. Imperial County transfers were continued due to the disproportionate number of patients with COVID-19, regional proximity, and no ECMO capabilities at their hospitals. During any phase, if an individual ECMO center was near their maximum census, all appropriate ECMO candidates were referred and transferred to other centers within the consortium with availability.

#### ECMO Reallocation Plan if All Consortium ECMO Resources Are Used

If all ECMO resources were used and additional patients required ECMO (COVID-19 or non-COVID-19), each ECMO center would assess all current patients on ECMO for predicted duration of support and survival. This would determine the appropriateness of continued ECMO support. For patients with COVID-19 initiated on ECMO, re-allocation of support will not be considered for 10 d if the patient remained stable. Once patients were identified for potential reallocation, an *ad hoc* ECMO consortium meeting was called.

During the *ad hoc* meeting, patient status and community resources are reviewed to determine that no other hospital system could provide further ECMO care. Next, the ECMO center notifies their internally established triage review committee that included risk management and the ethics committee. If ECMO withdrawal was decided after these 2 steps, then the patient’s surrogate decision maker was notified.

### Consortium Operations

#### Referral, Triage, and ECMO Consent Process

The referring medical provider would contact 1 or multiple ECMO centers through the institutional transfer center or by directly contacting the coordinator. The group messaging and consortium report, detailed prior, were crucial in real-time triaging of appropriate patients to the ECMO center with the most percent availability. This ECMO center with the most available capacity (accounting for staffing, equipment, and bed availability), would evaluate the patient and accept if appropriate.

If 2 centers were at the same percentage capacity with available staffing, equipment, and beds, then the patient would be sent to the insurance preferred center. If the insurance provider had no preference, or if the patient was unfunded, then the patient would be sent to the ECMO center with the least amount of stretched resources.

Funding and insurance were not discussed during ECMO candidacy evaluations. Insurance may have influenced the preferred center for transfer. If an insurance preferred center was unable to accept the patient, then a transfer agreement was established between the referring and ECMO center. For patients without funding, a transfer agreement was established between the referring and ECMO center.

If the surrogate requested that the patient be transferred to a particular ECMO center, that center would be contacted initially. If the preferred center did not have ECMO capacity, the family was informed that the patient would be referred to another center. However, if the patient was an appropriate ECMO candidate and the surrogate refused transfer to the alternate center, the ECMO coordinators would discuss possible transfers between centers to create bed availability. This process would be done on a case-by-case basis and was never implemented. Once the transfer process was initiated, the referring provider informed the surrogate decision-maker to be available for contact by the ECMO center.

#### Advance Transport Teams

One center within the consortium (Scripps Health) has an internally staffed advanced critical care transport team capable of transporting patients who were proned or receiving advanced ventilator settings or inhaled pulmonary vasodilators. Another center (University of California, San Diego Health – UC San Diego Health) has the ability to provide “mobile” ECMO to hospitals outside of their system for patients too unstable for transport. The UC San Diego Health mobile ECMO team traveled to the patient’s bedside, initiated ECMO, and then transferred to their ECMO center for continued care.

#### Consortium Communication, Meetings, and Reports

Continuous communication among consortium ECMO centers was established by having all ECMO coordinators on a group messaging application (WhatsApp, Facebook, California). Protected health-care information was not shared through this platform. The group messaging was updated in real time regarding each center’s ECMO census, urgent community ECMO needs, and if contingency or crisis phases were declared. Due to shared ECMO criteria, any single center could work up an ECMO referral independently and the decision to accept or decline was applied to all of the consortium centers. If 1 center was evaluating an ECMO referral, the other centers were notified to prevent simultaneous workups.

A consortium report was kept up to date with each center’s ECMO status (Table 2), on a HIPPA compliant cloud-based storage service (OneDrive, Microsoft). The report was shared with the San Diego County Health Department for continuous monitoring of the region’s ECMO census and needs.

**Table 2.** ECMO consortium outcomes from March 1 to November 31, 2020

	Total, n	Other information
San Diego ECMO capacity (based on equipment and staffing)	23	Maximum capacity 32^a^
Total ECMO runs for patients with COVID-19	97	
Equipment transfers between consortium centers	30	Six ECMO controllers, 10 cannulas, 1 oxygen blender, 11 oxygenators, 2 pump packs
Consortium multi-center meetings	54	Twenty-four weekly scheduled meetings; 30 ad hoc meetings concerning patient candidacy or management
ECMO patients received from outside consortium counties (San Diego and Imperial) or the state of California	10	One patient from Arizona; 2 patients from Riverside County
ECMO referrals sent to another center within the consortium due to capacity limitations	9	All 9 patients were placed on ECMO
ECMO referrals sent to another center within the consortium center due to insurance approval	1	Patient was placed on ECMO
Advanced critical care transports to an ECMO center	29	
Mobile ECMO	18	Provided to mobile ECMO from 7 referring hospitals in 3 California counties
Survival to hospital discharge	3-1-2020 to 11-31-2020	12-1-2020 to 4-30-2021^c^
Sharp HealthCare	^b^	^b^
Scripps Health	21.1% (7/33)	40% (4/10/6)^c^
UC San Diego Health	48.5% (16/33)	55% (11/20/4)^c^

a Maximum capacity with alternative staffing and limited operating room cases.

b Declined to provide.

c Survival to hospital discharge/total patients from 12-1-2020 to 4-30-2021/currently on ECMO.

The consortium met weekly by means of video conferencing (Zoom application, Zoom Video Communications). During the meeting, each center reported their COVID-19 census, mechanical ventilator use, ICU bed availability/census, ECMO patients, and potential ECMO candidates. Each center also reported their current ECMO capacity and any equipment or staffing issues. Due to multiple ECMO centers using the same perfusion staffing agencies, ECMO specialist staffing was discussed. Time was given during each weekly meeting to discuss challenging or unique clinical cases and patient management strategies.

In addition to the group messaging application and the weekly meetings, ad hoc meetings were called for urgent issues. For example, an ad hoc meeting was called in June of 2020, the first time San Diego County reached 80% of ECMO capacity and transitioned into crisis phase. The meeting was held to confirm the transition and to ensure that all centers were prepared for the implications (ie, changing ECMO criteria). Ad hoc meetings occurred when the medical team was unsure of appropriate ECMO candidacy due to relative contraindications and to determine reallocation of ECMO equipment or resources.

#### Equipment Sharing Between ECMO Centers

If required, ECMO equipment would be shared among centers to maximize the region’s ECMO census. Equipment would be requested directly between the centers through the ECMO coordinators using the group messaging application (WhatsApp, Facebook, California). The ECMO equipment company representatives and contracted perfusion companies also helped facilitate transfer of equipment between centers. Each center kept logs of both borrowed and lent equipment. The centers also rented ECMO equipment, which was further shared within the consortium.

## Results

**Figure 2.**
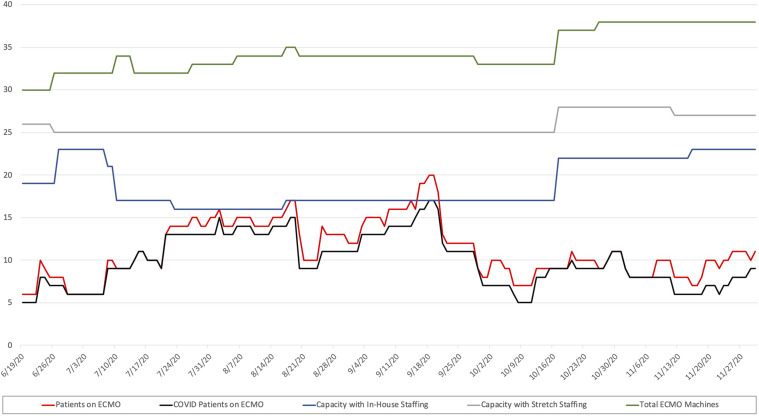
Daily ECMO census and capacity of San Diego County from June 19 to November 30, 2020. Data before June 19, 2020, was not tracked by the county health department.

From March 1 to November 30, 2020, the consortium centers placed 97 patients on ECMO (Table 2 and Figure 2). One patient was from a neighboring state and 8 were from Imperial County.

From June 19 to November 27, 2020, the consortium was in contingency and crisis phase 79% and 21% of the time, respectively (Figure 2). Pandemic ECMO phases data from March to May, 2020, were not collected. Reallocation of ECMO resources was never required. No patients were declined ECMO based on insurance or funding status.

ECMO equipment was moved between centers 30 times, as patient census fluctuated between locations, allowing the consortium to maximize capacity. Due to redistribution of potential ECMO patients from high (near maximum) census centers to lower census centers, 8 additional patients were ultimately able to receive ECMO. Twenty-nine patients referred for ECMO on high positive-end expiratory pressure requirements and inhaled vasodilators were transported by Scripps Health’s critical care transport team. The UC San Diego Health mobile ECMO team placed 18 patients on ECMO due to clinical instability. All of these patients were transferred to UC San Diego Health for continued care.

One patient on ECMO was transferred to the regional lung transplant center (UC San Diego Health) in the consortium. Three others were referred for lung transplant evaluation but were not candidates.

## Discussion

The establishment of the Southern California ECMO consortium allowed for: (1) equitable provision of ECMO to patients in our region; (2) 97 patients received ECMO from 3 California counties (San Diego, Imperial, and Riverside) and a neighboring state (Arizona); (3) sharing of ECMO expertise and equipment to increase overall county census; (4) efficiency in patient referrals; (5) ability to perform mobile ECMO and advanced critical care transport with subsequent distribution of patients to centers with capacity; and (6) re-allocation of ECMO resources never occurred.

The consortium’s initial goal was to provide ECMO fairly to patients in San Diego and Imperial Counties. However, during the pandemic, our consortium of ECMO centers received referrals from outside counties and states. While not in crisis phase, we evaluated and accepted appropriate ECMO candidates from outside the consortium’s region (ie, Riverside County, Arizona, Nevada). This was possible due to the consortium’s tracking of each ECMO center’s capacity and census.

There were multiple advantages to the consortium. The established ECMO criteria allowed for easy referral to other centers if 1 was at capacity or due to issues with insurance approval. Shared ECMO criteria and real-time communication improved efficiency by preventing concurrent evaluations from multiple centers. If a patient was unstable for conventional transfer to an ECMO center in our consortium, the advance transport teams (Scripps Health) or the UC San Diego Health mobile ECMO team would be notified to consider patient retrieval. Equipment and referral sharing increased the region’s maximum census and ensured that the ECMO burden did not disproportionally affect 1 center. Finally, the consortium shared ECMO expertise on mobilization, anticoagulation, and complications in multidisciplinary meetings.

Before the formation of the ECMO consortium, San Diego County was unable to determine county-wide ECMO capacity or use. A partnership between the ECMO consortium and the County of San Diego enables near real-time assessment of the region’s ECMO capacity. Through analysis of regional capacity, ECMO resources can be optimized and matched to patient and community needs. Early detection of evolving shortages alerts the San Diego County leadership to request additional resources. For example, while each center typically focused on equipment parameters (eg, number of ECMO machines), county level data made it clear that staffing was the limitation to increased ECMO capacity.

Future expansion of the consortium is anticipated and will likely persist after the COVID-19 pandemic. Our ECMO centers have received referrals from neighboring counties (Riverside, Orange, Los Angeles, and San Bernardino) and states (Arizona and Nevada), some of which have their own ECMO centers. Presumably, an expansion of our consortium would further facilitate rapid evaluation and match patients with available resources for an even larger region. Current discussions with Los Angeles and Orange Counties ECMO centers are in process to join the consortium. While our efforts have focused largely on veno-venous ECMO for ARDS, the infrastructure could be used for veno-arterial ECMO, which might require more rapid evaluation and dispatch of a mobile ECMO team. The consortium should allow us to evaluate ECMO outcomes at a regional level. Finally, the network provides an infrastructure to allow for modifications of ECMO inclusion/exclusion criteria and management as increasing evidence and/or new guidelines on COVID-19 are published.

## Conclusions

During the COVID-19 pandemic, our consortium provided equitable access to ECMO across multiple counties and states, and local collaboration increased our region’s maximum ECMO census. We believe this consortium model can be applied to other regions and will be essential for areas otherwise lacking ECMO resources or capacity.

## Supplementary material

For supplementary material accompanying this paper visit http://dx.doi.org/10.1017/dmp.2021.179. click here to view supplementary material
