# Supplementary material for: The Southern California Extracorporeal Membrane Oxygenation Consortium During the Coronavirus Disease 2019 Pandemic
Source: Disaster Med Public Health Prep. 2021 Jun 8:1–8. doi: 10.1017/dmp.2021.179 (PMC8314051; doi:10.1017/dmp.2021.179)
Supplement: Supplementary file 1 [file S1935789321001798sup001.docx]

Supplementary Appendix

This appendix has been provided by the authors to give readers additional information about their work.

|  | San Diego County | Tijuana, Mexico | Imperial County | Mexicali, Mexico |
| --- | --- | --- | --- | --- |
| Total Population | 3.3 million | 2.1 million | 170,957 | 1 million |
| Population Ranking | 2 of 58 in California |  | 30 of 58 in California |  |
| SARS-CoV-2 total cases (rate per 100,000), reported on November 30, 2020 | 83,421 (2,488.9 per 100,000) | 7,963 (379.2 per 100,000) | 16,364 (9080.2 per 100,000) | 12,342 (1234.2 per 100,000) |
| SARS-CoV-2 incidence rate on 12/1/2020 | 30.5/100,000 | 37.2/10,000 | 52.8/100,000 | Unknown |
| Centers with comprehensive ECMO capabilities (total hospitals) | 4 ^a^ (24) | 1 ^b^ (Unknown) | 0 (2) | 0 (Unknown) |

e-Table 1. Population and SARS-CoV-2 infection rate of southern California counties, neighboring regions in Mexico, and cities as of December 1^st^, 2020. ^a^ One center can provide ECMO only to pediatric patients. ^b^ Prior to the pandemic had no ECMO capabilities. ECMO, extracorporeal membrane oxygenation. ICU, intensive care unit. SARS-CoV-2, severe acute respiratory syndrome coronavirus 2.

| Do you have 24/7 capability to initiate ECMO? |
| --- |
| Do you transport patients out to an ECMO center once on ECMO? |
| How many total patients are currently on ECMO? |
| How many COVID-19 patients are currently on ECMO? |
| Total number of ECMO machines |
| What is your hospital TOTAL capacity for ECMO with current staffing? |
| What is your hospital STRETCH ECMO capacity (using additional contract staffing)? |
| How many Complete ECMO pumps are reserved as back up (for NON-COVID/emergent use) |
| How many patients are currently being watched for potential ECMO |
| Date Updated |

e-Table 2. Consortium ECMO report sections. ECMO, extracorporeal membrane oxygenation. COVID-19, coronavirus disease 2019.
